# Tendon dECM Composited with Chitosan with Loading Skin Precursor Stem Cell Exosome for Enhanced Diabetic Wound Healing

**DOI:** 10.3390/gels12050361

**Published:** 2026-04-26

**Authors:** Yunguang Chen, Yingying Liang, Yaling Deng, Lei Nie

**Affiliations:** 1 Department of Basic Medical Sciences, Wuxi School of Medicine, Jiangnan University Medical Center, Jiangnan University, Wuxi 214122, China; 2 College of Life Sciences, Xinyang Normal University, Xinyang 464000, China; liangyingying2468@126.com; 3 College of Intelligent Science and Control Engineering, Jinling Institute of Technology, Nanjing 211169, China; yalingdeng@jit.edu.cn

**Keywords:** exosome, hydrogel, diabetic wounds, angiogenesis

## Abstract

Diabetic wounds are a common and severe complication of diabetes mellitus, characterized by delayed healing due to persistent inflammation, impaired angiogenesis, and cellular dysfunction. Conventional therapeutic approaches remain limited in efficacy. In recent years, exosomes have attracted considerable attention in wound healing and regenerative medicine because of their crucial role in intercellular communication and tissue repair. However, rapid clearance of exosomes in vivo greatly limits their therapeutic efficacy. To address this critical limitation, we engineered a decellularized extracellular matrix (dECM)-based hydrogel system functionalized with exosomes derived from skin-derived precursor cells (SKPs). This biomimetic scaffold was designed to serve as a local exosome-delivery platform at the wound site, with the aim of improving exosome utilization and augmenting their regenerative effects. Comprehensive in vitro characterization demonstrated that the exosome-loaded composite hydrogels exhibited robust pro-angiogenic activity, as evidenced by enhanced endothelial cell proliferation, migration, and tube formation. Moreover, the hydrogels displayed significant antibacterial effects against wound-relevant pathogens and potent reactive oxygen species (ROS)-scavenging capacity, thereby mitigating oxidative damage. Notably, the composite hydrogels also promoted the phenotypic polarization of macrophages toward the pro-regenerative M2 phenotype. In parallel, in vivo studies using a streptozotocin-induced diabetic rat wound model confirmed that treatment with the composite hydrogels significantly accelerated wound closure rates compared to control groups. Histological and immunohistochemical analyses revealed enhanced angiogenesis, as evidenced by increased CD31-positive microvessel density, as well as improved collagen deposition, re-epithelialization, and an attenuated local inflammatory microenvironment characterized by reduced pro-inflammatory cytokine expression and elevated M2 macrophage infiltration. Collectively, the SKPs exosome-loaded dECM based composite hydrogels developed in this study represent a potential therapeutic strategy for the treatment of diabetic wounds.

## 1. Introduction

Diabetic wounds, a serious complication of diabetes mellitus, are marked by delayed healing processes resulting from hyperglycemia-induced cellular dysfunction, diminished angiogenesis, and persistent inflammation [1,2]. These wounds frequently result in extended hospital stays, heightened susceptibility to infections, and, in severe cases, amputations, thereby substantially affecting patients’ overall quality of life. According to epidemiological data, approximately 15–25% of individuals with diabetes will develop foot ulcers at some point in their lives, placing a considerable financial burden on healthcare systems globally [3]. However, traditional treatments have limited effectiveness, and new therapeutic strategies need to be sought to improve the repair of diabetic wounds.

Exosomes, as key mediators of intercellular communication, regulate cellular functions and tissue homeostasis by transferring bioactive molecules, including proteins, lipids, and nucleic acids. In recent years, exosomes have attracted significant attention in the fields of skin repair and regenerative medicine, demonstrating immense potential for application. Studies have found that exosomes can promote wound healing by modulating the activity of fibroblasts and keratinocytes [4]. Additionally, exosomes can enhance wound healing by regulating immune cell function, particularly demonstrating outstanding efficacy in the treatment of diabetic and chronic wounds [4,5]. For example, adipose-derived stem cell exosomes (ADSC-exos) have been proven to accelerate wound healing by modulating inflammatory responses and promoting cell proliferation, migration, and angiogenesis [6]. Epidermal stem cell (ESC)-derived exosomes enhance the proliferation and migration of diabetic fibroblasts and macrophages, and promote M2 macrophage polarization [7]. Human endothelial progenitor cell-derived exosomes (EPC-EXO) can promote diabetic wound healing by regulating vascular endothelial cell function [8]. Skin-derived precursor cells (SKPs), as a novel type of stem cell originating from the dermis, possess robust self-renewal capacity, multipotent differentiation potential, and low immunogenicity. These characteristics make SKPs an ideal candidate for treating diabetic wounds and other chronic skin injuries [9]. Exosomes derived from SKPs combine the multipotent differentiation potential of SKPs with exosomes’ efficient delivery capabilities, offering unique advantages for accelerating wound healing and tissue regeneration. They demonstrate significant potential for application in the treatment of diabetic wounds.

Current exosome-based therapeutic strategies, despite their considerable promise in regenerative medicine, face several critical limitations that hinder their clinical translation. One of the most significant challenges is the rapid clearance of exogenously administered exosomes from the wound microenvironment following local injection. This accelerated elimination—mediated by lymphatic drainage, interstitial fluid flow, and phagocytic uptake by resident immune cells—results in a markedly short biological half-life and diminished bioavailability at the target site. Consequently, achieving sustained therapeutic effects often necessitates frequent and repeated administrations, which not only compromises patient compliance but may also exacerbate local tissue trauma, provoke additional inflammatory responses, and disrupt the fragile healing process. To overcome these inherent limitations, the integration of exosomes with biomaterial-based delivery systems has emerged as a promising strategy. By conjugating, encapsulating, or immobilizing exosomes within biocompatible scaffolds—such as hydrogels, porous sponges, or electrospun nanofibrous matrices—their retention time within the wound bed can be substantially prolonged. This localized sequestration protects exosomes from premature degradation and washout while supporting local availability of their bioactive cargo (including proteins, lipids, and microRNAs) directly at the injury site. Such biomaterial-assisted delivery not only enhances the therapeutic efficacy of exosomes by maintaining optimal local concentrations over clinically relevant time frames but also minimizes systemic off-target effects. Collectively, this synergistic approach addresses the pharmacokinetic shortcomings of conventional exosome administration and maximizes their regenerative potential in wound healing applications [10,11].

Chitosan possesses functional properties, including antibacterial activity, non-toxicity, ease of modification, and biodegradability. Through chemical modification, chitosan derivatives with additional biomedical functions can be generated, thus demonstrating great potential in drug delivery systems, tissue regeneration, and antibacterial applications [12,13]. dECM possesses excellent biocompatibility, low immunogenicity, and highly biomimetic extracellular matrix characteristics. Its retained natural ECM components provide an ideal microenvironment for cell adhesion, migration, and proliferation, thereby promoting tissue reconstruction and regeneration. Our previous studies have confirmed the favorable biosafety and in vivo compatibility of this dECM material, providing a solid foundation for its further application in wound repair [14]. Through the integration of dECM and chitosan, a composite biomaterial system with both biomimetic structural support and functional regulatory capabilities can be constructed. On this basis, combining exosomes with a chitosan polymer can optimize the exosome delivery system, providing a microenvironment conducive to their survival [15,16]. This approach prevents their rapid degradation or clearance in vivo, thereby prolonging their localized action at the wound site [17,18,19]. This combined strategy not only reduces the frequency of administration but also minimizes secondary tissue damage while significantly enhancing therapeutic outcomes. It provides a more efficient and safer treatment option for wound healing [20].

In this study, we developed a multifunctional dECM@QCS composite hydrogel loaded with SKP-derived exosomes for diabetic wound repair. The rationale of this design was to integrate the biomimetic structural support of dECM, the antibacterial and functional properties of QCS, and the regenerative bioactivity of SKP-derived exosomes into a single platform. We further evaluated whether this integrated system could improve the diabetic wound microenvironment through antibacterial, antioxidative, immunomodulatory, and pro-angiogenic effects, thereby promoting tissue regeneration and wound healing [21].

## 2. Results and Discussion

### 2.1. Isolation and Characterization of Exosomes

**Figure 1:**
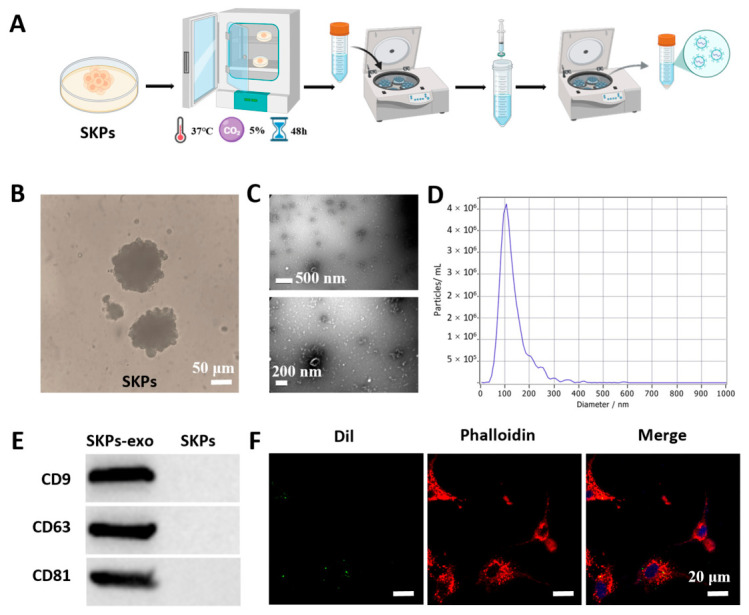
Isolation and characterization of exosomes. (**A**) Schematic diagram of the extraction process of SKP-derived exosomes. (**B**) Observation of the morphology of primary cultured SKPs using optical microscopy (scale = 50 μm). (**C**) Morphological observation of exosomes using transmission electron microscopy (TEM). (**D**) Nanoparticle tracking analysis (NTA) was used to detect the particle size distribution of exosomes. (**E**) The expression of marker proteins (CD9, CD63, and CD81) on the surface of exosomes was detected by Western blot. (**F**) Immunofluorescent staining (Dil/Phalloidin staining) was used to detect the uptake of exosomes.

SKP-derived exosomes were isolated according to the schematic workflow as shown in Figure 1A. A three-dimensional (3D) suspension culture system of SKPs was successfully in vitro established after the SKPs were isolated and cultivated from Sprague-Dawley (SD) rats. Under microscopic observation, the typical SKPs spheroids were clearly observed, and the cells displayed the compact spherical aggregates with clear boundaries (Figure 1B). Subsequently, exosomes were isolated and enriched from the culture supernatant of SKPs using ultracentrifugation, yielding highly purified SKP-derived exosomes. Transmission electron microscopy (TEM) images revealed that the isolated vesicles exhibited the characteristic cup-shaped morphology of exosomes (Figure 1C). In addition, nanoparticle tracking analysis (NTA) further demonstrated that the particle size of SKP-derived exosomes was mainly distributed between 30 and 150 nm (Figure 1D), consistent with the typical exosome size range (40–160 nm) [22,23]. Western blot (WB) analysis confirmed strong positive expression of the exosomal markers CD9, CD63, and CD81 (Figure 1E). In addition, fluorescently labeled exosomes were co-cultured with endothelial cells, and distinct green fluorescent signals were observed in the cytoplasm under a fluorescence microscope. These signals did not overlap with nuclear staining, indicating that the exosomes were effectively internalized by endothelial cells rather than merely adsorbed on the cell membrane surface (Figure 1F). Collectively, the morphological characteristics, particle size distribution, marker protein expression, and cellular uptake experiments confirmed the successful isolation and identification of SKPs’ exosomes.

### 2.2. Fabrication and Characterization of dECM@QCS Composite Hydrogels

**Figure 2:**
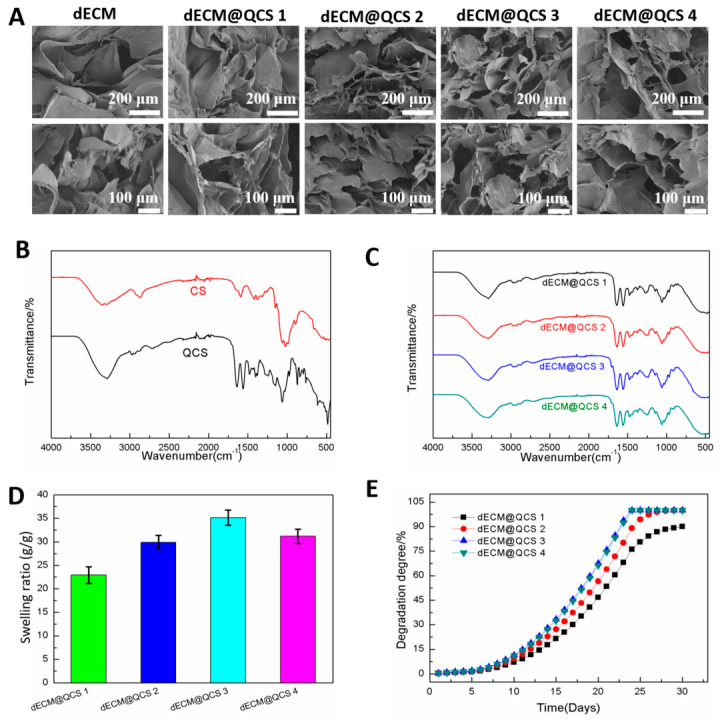
Synthesis and characterization of dECM@QCS composite hydrogels. (**A**) Scanning electron microscope (SEM) images of dECM@QCS composite hydrogels with different dECM/QCS ratios. (**B**) Fourier transform infrared spectroscopy (FTIR) spectra of CS and QCS. (**C**) FTIR spectra of dECM@QCS composite hydrogels. (**D**) Swelling ratios of dECM@QCS composite hydrogels measured in PBS. (**E**) In vitro degradation curves of dECM@QCS composite hydrogels investigated in PBS.

The dECM was synthesized using the fresh bovine Achilles tendon tissue using the typical approach according to the previous reports [24,25]. Scanning electron microscopy (SEM) images show that the pure dECM hydrogel has a typical polymer-porous structure, providing a strong structural basis for subsequent cross-linking with chitosan derivatives, quaternized ammonium chitosan (QCS) (Figure 2A). Then, the dECM@QCS composite hydrogel was fabricated by compositing dECM with QCS polymer, and EDC/NHS were added to facilitate the initial crosslinking. The fabricated dECM@QCS composite hydrogels displayed the interconnected microstructure as the pure dECM hydrogel. Compared to the pure dECM hydrogel, the composite hydrogels demonstrated a slight regular micro-pore structure. However, most of the pores in hydrogels were irregular; thus, the pore size distribution was not displayed in this work [24]. Next, the Fourier transform infrared spectroscopy (FT-IR) was used to confirm the successful synthesis of QCS, and the interactions between QCS and dECM in the dECM@QCS composite hydrogels were also investigated. QCS could be facilely synthesized via grafting cationic quaternary ammonium groups on chitosan [26,27]. According to the FT-IR spectra of chitosan and QCS, a strong and broad -C-N^+^ stretching vibration absorption band at 1200–700 cm^−1^, was observed, confirming the successful introduction of quaternary ammonium groups (Figure 2B). After compositing dECM with QCS, it was found that the broad and strong peak of carboxylic acid O-H in the dECM@QCS composite hydrogels disappeared in the FT-IR spectra of hydrogels (Figure 2C). At approximately 1640–1680 cm^−1^, a very strong and sharp amide I band absorption peak was observed, and at approximately 1500–1560 cm^−1^, a strong amide II band absorption peak was observed, proving that the amide connection was formed between dECM and QCS in the dECM@QCS composite hydrogels.

Considering that the water absorption capacity and degradation ability are important parameters for hydrogel dressing in wound healing applications [28,29]. The swelling ratios of the dECM@QCS composite hydrogels were measured in phosphate-buffered saline (PBS). The water absorption rate of dECM@QCS was influenced by the addition of dECM content (Figure 2D). Due to the highly hydrophilic properties of dECM, the increase in dECM content could increase the hydrophilicity of dECM@QCS composite hydrogels, resulting in greater water absorption capacity. However, as the dECM content continues to increase, the addition of large amounts of dECM will weaken the hydrogel’s network, resulting in a slight decrease on water absorption performance. In addition, all hydrogels exhibit excellent in vitro degradation performance, with degradation essentially complete within 20 days (Figure 2E). All dECM@QCS composite hydrogels displayed similar degradation profiles.

### 2.3. Cytocompatibility and Antibacterial Ability of dECM@QCS Composite Hydrogels

**Figure 3:**
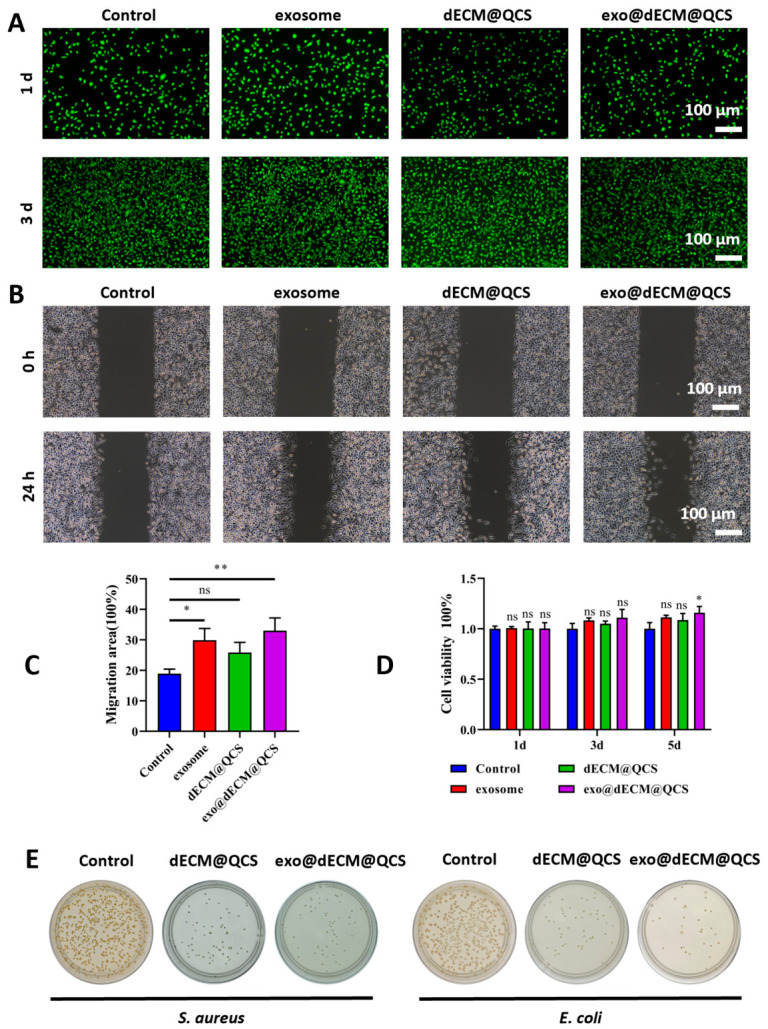
The cytocompatibility and antibacterial activity evaluation of the dECM@QCS and exo@dECM@QCS composite hydrogels. (**A**) Live cell staining was used to investigate the fibroblast proliferation (Scale = 100 μm). (**B**) Cell scratch assay was used to investigate the fibroblast migration (Scale = 100 μm). (**C**) Quantitative analysis results of the cell scratch experiment. (**D**) The CCK-8 experiment was used to detect the activity of fibroblasts. (**E**) The antibacterial properties of different materials were detected by plate counting method. “ns” indicates no statistical difference, * *p* < 0.05, and ** *p* < 0.01.

The cytocompatibility and antibacterial ability of the dECM@QCS composite hydrogels were evaluated. First, the cytocompatibility of the dECM@QCS composite hydrogels was investigated by culturing with fibroblasts cells (NIH 3T3 cells). After fibroblasts cells were cultured with hydrogel extracts for 1 day and 3 days, the live/dead staining (Calcein-AM and propidium iodide) was performed (Figure 3A). Fluorescence microscopy revealed that cells across all experimental groups predominantly exhibited green fluorescence, indicative of intact plasma membranes and metabolic activity. In contrast, the proportion of red-fluorescent, non-viable cells remained exceptionally low. Furthermore, the adherent fibroblasts displayed typical spindle-shaped morphology and well-spread cytoplasmic extensions, suggesting robust cell adhesion, proliferation, and a healthy physiological state. Collectively, these findings demonstrate that both the exosome-functionalized matrix and the dECM@QCS hydrogel possess excellent cytocompatibility and negligible in vitro cytotoxicity, thereby establishing a strong foundation for their potential application in tissue regeneration and repair.

The wound healing potential of the hydrogel formulations was quantitatively assessed using an in vitro scratch assay (Figure 3B,C). The results demonstrated that the exo@dECM@QCS composite hydrogel markedly enhanced the migratory capacity of fibroblasts compared to both the dECM@QCS hydrogels and the exosome-supplemented group when administered individually. Notably, the observed enhancement suggests a potential synergistic effect resulting from the functional integration of exosomes within the dECM@QCS matrix. Exosomes are nanoscale extracellular vesicles rich in a diverse array of bioactive macromolecules, including signaling proteins, lipids, and microRNAs (miRNAs) [22,30]. These molecular constituents are known to modulate key intracellular signaling pathways governing cytoskeletal reorganization, focal adhesion turnover, and directional cell migration. Concurrently, the dECM component serves as a biomimetic scaffold that recapitulates key physicochemical and biochemical attributes of the native tissue microenvironment, thereby supporting cell adhesion, spreading, and motility. Consequently, the combinatorial delivery of exosomes within a dECM-based hydrogel may potentiate cell migration through the convergence of biochemical cues from exosomes and biophysical support from the dECM matrix. This integrated biofunctional platform thus holds significant promise for accelerating wound closure and enhancing tissue regeneration.

The resultsCell viability was further evaluated using the Cell Counting Kit-8 (CCK-8) assay at 1, 3, and 5 days to assess the cytocompatibility of the materials. The results (Figure 3D) showed that no significant differences in cell viability were observed among the four groups at days 1 and 3. However, by day 5, the exo@dECM@QCS group exhibited higher cell viability than the other groups, indicating that the composite scaffold could better support cell activity over time and promote cell proliferation. These results suggest that the exo@dECM@QCS scaffold possesses good cytocompatibility and provides a favorable microenvironment for cell growth.

Previous studies have shown that quaternized chitosan and its derivatives generally exhibit antibacterial activity against both Gram-positive and Gram-negative bacteria, which is beneficial for preventing wound infection, particularly in chronic wounds such as diabetic ulcers [31]. In this study, the antibacterial properties of the materials were further investigated. The results (Figure 3E) showed that the dECM@QCS composite scaffold effectively inhibited the growth of Staphylococcus aureus and Escherichia coli. The antibacterial performance of the exo@dECM@QCS group was overall comparable to that of the dECM@QCS group, with no significant difference observed. This effect may be mainly attributed to the positively charged structure of quaternized chitosan, which can disrupt bacterial cell membranes and thereby inhibit bacterial growth. The antibacterial activity of the scaffold may help reduce the risk of wound infection and provide a more favorable environment for subsequent tissue repair.

### 2.4. Exo@dECM@QCS Regulates Macrophage Polarization and Oxidative Stress

**Figure 4:**
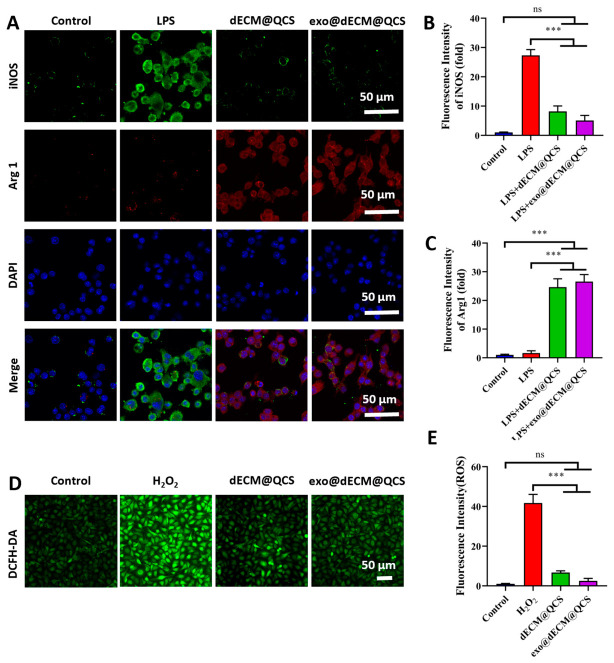
The macrophage polarization and oxidative stress regulated by the dECM@QCS and exo@dECM@QCS composite hydrogels. (**A**) Immunofluorescence staining was used to detect the expression of M1-type marker iNOS (green) and M2-type marker Arg1 (red) in macrophages. DAPI (blue) was used to label the cell nuclei (Scale = 50 μm). (**B**) Quantitative analysis results of iNOS fluorescence intensity. (**C**) Quantitative analysis results of Arg1 fluorescence intensity. (**D**) The intracellular ROS levels in HUVECs were detected by DCFH-DA staining (Scale = 50 μm). (**E**) Quantitative analysis results of ROS fluorescence intensity. “ns” indicates no statistical difference, and *** *p* < 0.001.

It is well known that chronic diabetic wounds are typically characterized by the persistent activation of M1-type macrophages and a sustained pro-inflammatory microenvironment, which hinder granulation tissue formation and epithelial regeneration. During diabetic wound repair, promoting macrophage polarization from the M1 phenotype toward the M2 phenotype is considered an important mechanism for regulating inflammation and facilitating tissue regeneration. M2 macrophages can secrete various anti-inflammatory and tissue repair-related cytokines, thereby alleviating excessive inflammatory responses and promoting wound healing [32]. In this study, immunofluorescence staining was performed to detect the expression of the characteristic markers iNOS (green) and Arg1 (red) in macrophages from each group to evaluate M1/M2 macrophage polarization (Figure 4A). The results (Figure 4B,C) showed that the dECM@QCS material reduced macrophage polarization toward the pro-inflammatory M1 phenotype while promoting their transition toward the M2 phenotype, suggesting its potential anti-inflammatory effect and beneficial role in tissue repair. This effect may be related to the ECM-like microenvironment provided by dECM and the favorable biological activity of quaternized chitosan, which together create suitable conditions for regulating macrophage function. Furthermore, the incorporation of exosomes further enhanced this effect and improved the material’s ability to promote M2 macrophage polarization, consistent with previous studies [33]. Exosomes are rich in various miRNAs, proteins, and signaling molecules that can regulate inflammation-related pathways and thus contribute to immune modulation and tissue repair.

In addition, the results demonstrated that the exo@dECM@QCS composite scaffold significantly reduced intracellular reactive oxygen species (ROS) levels and alleviated oxidative stress-induced damage (Figure 4D). Under inflammatory stimulation, excessive ROS production can disrupt cellular function and aggravate inflammatory responses, which is one of the major factors that hinder diabetic wound healing. The results (Figure 4E) showed that the ROS fluorescence signal was markedly reduced in the dECM@QCS group, while a further decrease was observed in the exo@dECM@QCS group, indicating that the composite scaffold possessed favorable antioxidant activity. This effect may be mainly attributed to the antioxidant and anti-inflammatory bioactivity of QCS, while the antioxidant regulatory factors carried by exosomes may further enhance the ROS-scavenging capacity of the composite system. The combined action of these components may help reduce excessive ROS accumulation and alleviate oxidative stress, thereby creating a more favorable microenvironment for cell growth and tissue repair. Overall, these findings suggest that the exo@dECM@QCS composite scaffold has potential advantages in regulating inflammatory responses and alleviating oxidative stress, supporting its possible application in chronic wound repair.

### 2.5. Exo@dECM@QCS Regulates Endothelial Cell Proliferation, Migration and Angiogenesis

**Figure 5:**
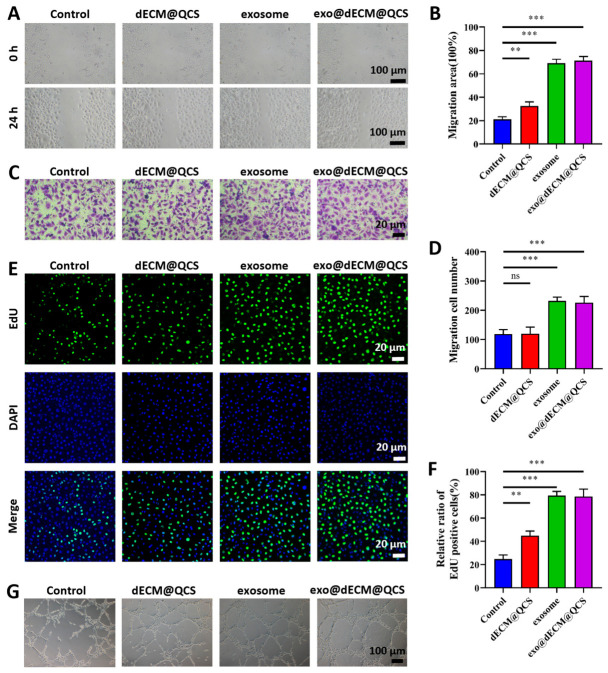
The endothelial cell proliferation, migration, and angiogenesis regulated by SKP-Exos and composite hydrogels. (**A**) Cell scratch assay was used to investigate the effects of different materials on the migration of endothelial cells (Scale = 100 μm). (**B**) Quantitative analysis results of the cell scratch experiment. (**C**) Transwell migration assay is used to detect the migration ability of endothelial cells (Scale = 100 μm). (**D**) Quantitative analysis results of the Transwell migration experiment. (**E**) Immunofluorescence staining was used to detect the proliferation of EdU-positive cells (Scale = 20 μm). (**F**) Quantitative analysis results of EdU-positive cells. (**G**) Representative images of the tubular formation experiment (Scale = 100 μm). “ns” indicates no statistical difference, ** *p* < 0.01, and *** *p* < 0.001.

Angiogenesis is a crucial process in tissue repair and regeneration, as it provides oxygen and nutrients to damaged tissues and supports new tissue formation. Previous studies have shown that exosomes can regulate endothelial cell proliferation, migration, and angiogenesis-related signaling pathways during wound healing, thereby promoting angiogenesis [34]. In this study, we further investigated the in vitro effects of the exo@dECM@QCS composite scaffold on endothelial cell function. During angiogenesis, endothelial cell migration is an essential step in the sprouting of new blood vessels from pre-existing vasculature. In the scratch assay, increased migration was observed in all groups 24 h after scratching (Figure 5A,B). Among these groups, the exosome group showed a significantly reduced scratch width, while the exo@dECM@QCS group exhibited the narrowest scratch width and the highest migration area ratio among all groups. Consistent with these findings, the Transwell migration assay showed that the number of migrated cells in the exo@dECM@QCS group was significantly higher than that in the other three groups (Figure 5C,D). These results indicate that the exosome-loaded dECM@QCS composite scaffold can effectively enhance endothelial cell migration. This effect may be related to the bioactive molecules carried by exosomes, such as miRNAs, proteins, and growth factors, as well as the ECM-like microenvironment provided by dECM, which supports cell adhesion and migration.

Furthermore, EdU staining was performed to evaluate cell proliferation by determining the proportion of EdU-positive cells in each group (Figure 5E,F). The results showed that the exo@dECM@QCS group exhibited the highest proportion of EdU-positive cells, which was significantly greater than that in the other three groups, indicating that the composite scaffold loaded with exosomes more effectively promoted endothelial cell proliferation. Since endothelial cell proliferation is essential for maintaining the angiogenic process, this effect may also contribute to the enhanced pro-angiogenic activity of the composite scaffold.

In addition, an in vitro tube formation assay was conducted to evaluate the effect of exo@dECM@QCS on angiogenic behavior (Figure 5G). The results showed that endothelial cells treated with exo@dECM@QCS formed tubular structures with greater total length, more branches, and increased numbers of closed loops, indicating enhanced angiogenic activity. This effect may be attributed to the regulatory role of exosomes in angiogenesis-related pathways and the supportive three-dimensional microenvironment provided by dECM, which together facilitate vascular network formation.

Overall, these results suggest that the exo@dECM@QCS composite scaffold can promote endothelial cell migration, proliferation, and angiogenic behavior in vitro, supporting its potential application in vascular regeneration and wound healing.

### 2.6. Exo@dECM@QCS Promotes Wound Healing in Diabetic Rats

Wound healing is a complex and highly coordinated biological process that generally consists of four stages: hemostasis, inflammation, proliferation, and remodeling. Among these stages, timely regulation of inflammation and effective initiation of angiogenesis are crucial for successful wound repair. In chronic wounds such as diabetic wounds, persistent inflammation and insufficient angiogenesis often lead to delayed healing. Therefore, regulating the inflammatory microenvironment and promoting neovascularization are considered important strategies for accelerating chronic wound repair. Previous studies have shown that exosomes can promote wound healing by regulating immune responses, facilitating macrophage polarization, and enhancing endothelial cell migration and angiogenesis [35]. In addition, biomaterials that provide a natural extracellular matrix (ECM)-like microenvironment can support cell adhesion, growth, and tissue regeneration, thereby further promoting the wound healing process.

**Figure 6:**
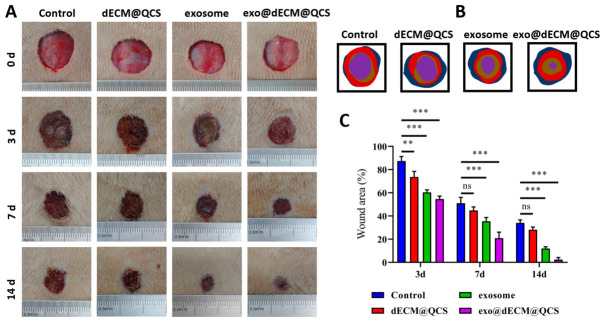
The in vivo experimental of SKP-Exos and composite hydrogels on promoting skin wound healing. (**A**) Photos of skin wounds in rats at different time points (0, 3, 7, and 14 days). (**B**) Dynamic analysis of wound healing process. (**C**) Quantitative analysis results of the wound area in rats. “ns” indicates no statistical difference, ** *p* < 0.01, and *** *p* < 0.001.

To evaluate the in vivo therapeutic effect of exo@dECM@QCS, a diabetic full-thickness skin wound model was established. The wounds were then treated with exosomes, dECM@QCS, or exo@dECM@QCS. Photographs of the wounds were taken at specific time points, and wound areas were measured to assess the healing process (Figure 6A). The results (Figure 6B,C) showed that both the dECM@QCS group and the exosome group promoted wound healing to some extent compared with the control group, whereas the exo@dECM@QCS group exhibited a significantly faster wound closure rate than the other groups. As healing progressed, wounds in the exo@dECM@QCS group showed more rapid contraction and closure, indicating an improved therapeutic effect.

These findings suggest that the exosome-loaded dECM@QCS composite scaffold can effectively promote the repair of diabetic skin wounds. This effect may be related to the combined actions of the biomaterial scaffold and exosomes. Specifically, the dECM@QCS scaffold provides an ECM-like microenvironment that supports cell adhesion and tissue regeneration, while exosomes may contribute to wound repair by regulating inflammation and promoting angiogenesis. Together, these effects may help improve the wound microenvironment and accelerate the healing process. Overall, these results suggest that exo@dECM@QCS has potential as a therapeutic strategy for diabetic chronic wounds.

**Figure 7:**
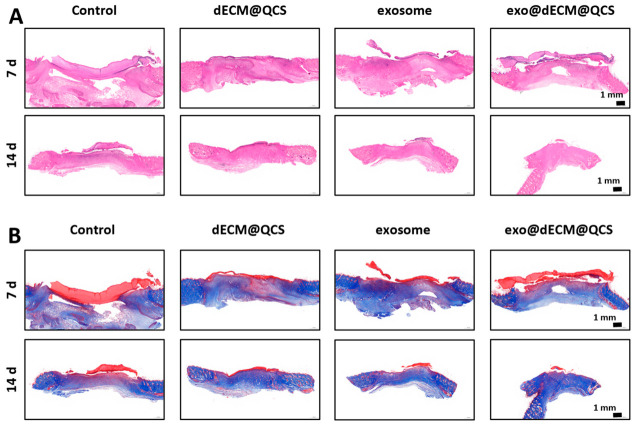
Histological analysis of skin wounds in the diabetic rat using different treatments. (**A**) The H&E staining images of skin wound in each group at 7 days and 14 days, Scale = 1 mm. (**B**) The Marson’s trichrome staining images of skin wound healing in each group at 7 days and 14 days (red represents muscle tissue, blue represents collagen fibers).

The wound healing effect was further evaluated by histological analysis using H&E staining and Masson’s trichrome staining. H&E staining showed that at day 7 post-operation, the exo@dECM@QCS group exhibited a more complete epithelial layer and a higher degree of epithelial regeneration than the other groups (Figure 7A). By day 14, the wounds in this group had achieved nearly complete epithelialization, and the epidermal structure appeared more continuous and intact, gradually resembling that of normal skin. In contrast, although the dECM@QCS group and the exosome group also showed improvement in epithelial regeneration, their effects remained less pronounced than those observed in the exo@dECM@QCS group, while the control group showed the slowest epithelial repair.

Masson’s trichrome staining further demonstrated that at day 7 post-operation, the exo@dECM@QCS group exhibited the most prominent deposition of newly formed collagen, with collagen fibers arranged in a relatively regular pattern (Figure 7B). By day 14, as healing progressed, the collagen fibers in this group became denser and more orderly, gradually approaching the collagen structure observed in normal skin tissue. In comparison, the other groups showed less collagen deposition and relatively loose and disorganized collagen arrangement.

These findings indicate that exo@dECM@QCS can promote granulation tissue formation, epithelial regeneration, and collagen matrix reconstruction in diabetic wounds. The enhanced healing effect may be related to the combined action of exosomes and the biomaterial scaffold. On the one hand, the dECM@QCS composite scaffold provides a three-dimensional microenvironment similar to the natural extracellular matrix, which supports cell adhesion, migration, and proliferation. On the other hand, exosomes contain various bioactive molecules, including miRNAs, proteins, and growth factors, which may help regulate inflammatory responses, promote angiogenesis, and enhance intercellular communication, thereby improving the wound healing microenvironment in diabetic wounds. Together, these effects may contribute to early granulation tissue formation and more orderly collagen remodeling during the later stages of healing.

Furthermore, these results are consistent with previous studies reporting the role of mesenchymal stem cell (MSC)-derived extracellular vesicles in skin wound healing, which have shown that exosomes can promote epithelial regeneration, enhance collagen deposition, and improve tissue structure during the repair process [36]. Therefore, the exo@dECM@QCS composite system shows potential for the treatment of diabetic chronic wounds.

### 2.7. Exo@dECM@QCS Synergistically Promotes Angiogenesis and Tissue Remodeling in Diabetic Wounds

**Figure 8:**
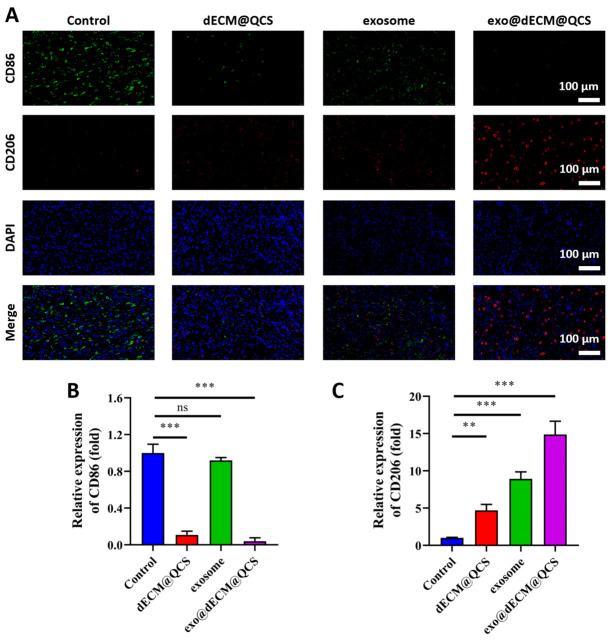
The effects of different treatment groups on the M1/M2 polarization of macrophages at day 3 after in vivo evaluation. (**A**) The immunofluorescent staining images for each group. CD86 (green) is a marker for M1-type macrophages, CD206 (red) is a marker for M2-type macrophages, and DAPI (blue) is used to label the cell nuclei (Scale = 100 μm). (**B**) Quantitative analysis of the relative expression of CD86 in each group. (**C**) Quantitative analysis of the relative expression of CD206 in each group. “ns” indicates no statistical difference, ** *p* < 0.01, and *** *p* < 0.001.

This study further evaluated the anti-inflammatory effect of the material by examining immune phenotype markers. The results showed that in the exo@dECM@QCS group, the pro-inflammatory macrophage marker CD86 (green) was barely detectable and its expression level was significantly lower than those in the other three groups, whereas the anti-inflammatory and repair-related marker CD206 (red) showed markedly enhanced positive expression, with levels significantly higher than those of the other groups (Figure 8A–C). These findings suggest that exo@dECM@QCS can promote macrophage polarization toward an anti-inflammatory, repair-associated phenotype, thereby contributing to a more favorable immune microenvironment for tissue repair.

In chronic wounds, persistent inflammatory responses are a major factor that impedes healing. The results of this study suggest that exo@dECM@QCS may help improve the local inflammatory microenvironment by promoting macrophage polarization toward a reparative phenotype, thereby helping to suppress excessive inflammation and support subsequent tissue regeneration. This effect may be related to the combined action of bioactive molecules carried by exosomes, such as miRNAs and proteins, together with the dECM@QCS scaffold, which provides a biomimetic extracellular matrix-like environment conducive to cell adhesion and immune regulation. Overall, these results indicate that exo@dECM@QCS has potential anti-inflammatory and pro-repair effects during chronic wound healing, consistent with previous studies [37].

**Figure 9:**
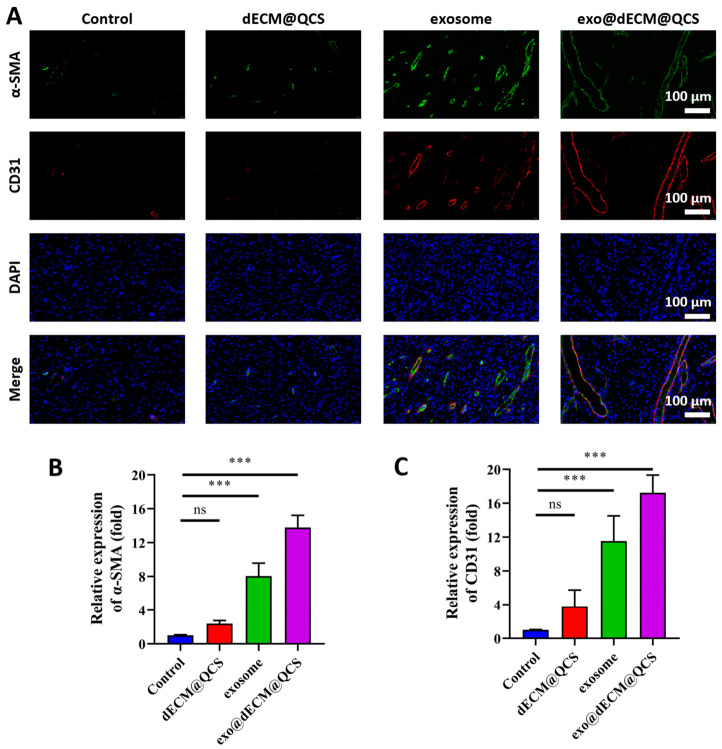
The effects of different treatment groups on the expression of angiogenesis-related proteins during skin wound healing at day 14. (**A**) The immunofluorescence staining images of α-SMA (green, actin, marking mature vascular smooth muscle cells) and CD31 (red, marking vascular endothelial cells) in the skin tissue. DAPI (blue) was used to mark the cell nuclei, and Merge was the three-color channel superimposed image (Scale = 100 μm). (**B**) Quantitative analysis of the relative expression of α-SMA in each group. (**C**) Quantitative analysis of the relative expression of CD31 in each group. “ns” indicates no statistical difference, and *** *p* < 0.001.

In addition, this study employed α-SMA and CD31 immunofluorescence staining to evaluate the formation of new blood vessels (Figure 9A). The results showed that in the exo@dECM@QCS group, the α-SMA fluorescence signal was the strongest, and more vascular smooth muscle-associated structures were observed, suggesting enhanced vascular maturation and wound contraction. At the same time, the angiogenesis-related marker CD31 also showed relatively high expression in this group, with more continuous endothelial structures observed. Quantitative analysis further supported these findings (Figure 9B,C), indicating that the composite scaffold promoted angiogenesis during wound healing.

**Figure 10:**
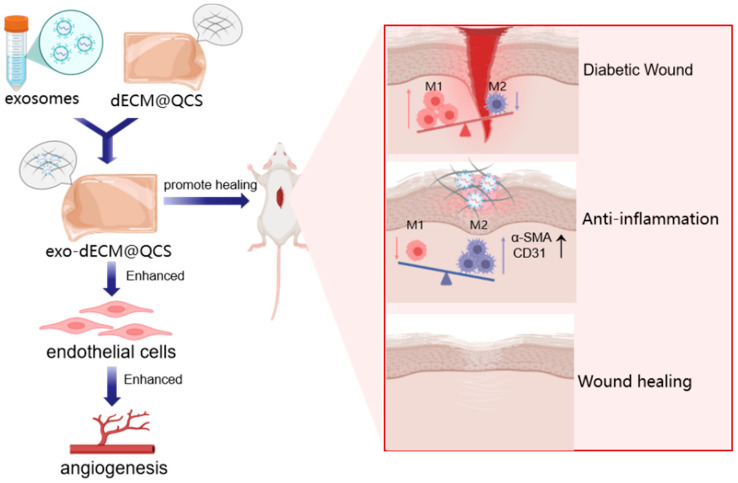
The schematic diagram illustrates the mechanism of exo@dECM@QCS composite hydrogels in promoting the healing of diabetic skin wounds.

Collectively, these findings suggest that the exo@dECM@QCS composite hydrogel may promote chronic wound healing through both immunomodulatory and pro-angiogenic effects. On the one hand, it may help improve the local immune microenvironment by reducing excessive inflammatory responses and promoting a shift toward a more repair-associated macrophage phenotype. On the other hand, it may enhance angiogenesis, as indicated by the increased expression of vascular-related markers observed in the wound tissue (Figure 10). Together, these effects may contribute to improved tissue regeneration and extracellular matrix remodeling during wound healing. Therefore, the exo@dECM@QCS composite hydrogel shows potential as a therapeutic candidate for the treatment of diabetic chronic wounds.

## 3. Conclusions

In the present study, a composite hydrogel system consisting of dECM integrated with QCS and loaded with skin-derived precursor exosomes (SKP-exos) was successfully fabricated. The in vitro biological evaluation revealed that the exo@dECM@QCS composite hydrogels significantly enhanced key cellular functions of endothelial cells, including proliferation, directional migration, and angiogenic tube formation, all of which are critical for neovascularization in regenerating tissues. Furthermore, in vivo experiments in a diabetic wound model demonstrated that exo@dECM@QCS markedly promoted re-epithelialization, augmented collagen deposition, and facilitated extracellular matrix remodeling, collectively leading to improved healing quality. In parallel, a notable reduction in local inflammatory infiltration was observed, which contributed to accelerated wound closure and more organized tissue architecture. These findings collectively suggest that the incorporation of SKP-exos into the dECM@QCS matrix holds substantial promise as a potential therapeutic strategy for the management of diabetic refractory wounds. Despite these encouraging outcomes, several limitations of the current study warrant acknowledgment. For instance, the optimization of key physicochemical properties of the dECM@QCS scaffold, such as pore architecture (size, interconnectivity, and porosity), degradation kinetics, and overall biocompatibility, requires further systematic investigation. Achieving an ideal synergy between the exosome cargo and the hydrogel matrix is essential to enable sustained release kinetics and preserve the bioactivity of exosomal contents over a clinically relevant time frame. Future studies should therefore focus on fine-tuning these parameters to maximize the regenerative potential of the exosome-functionalized matrix.

## 4. Materials and Methods

### 4.1. Chemicals

Chitosan (CS, 100–200 mPa·s of viscosity, deacetylation degree (DD) ≥ 95%), 1-(3-dimethylaminopropyl)-3-ethylcarbodiimide hydrochloride (EDC·HCl, 98%), N-Hydroxysuccinimide (NHS, 98%), 2’,7’-dichlorodihydrofluorescein diacetate (DCFH-DA, 97%), 2’,7’-dichlorodihydrofluorescein diacetate (DCFH-DA, 97%), glycidyltrimethylammonium chloride (GTMAC, >95%), sodium dodecyl sulfate (SDS), and pepsin (porcine origin, 1:3000) were purchased from Aladdin Co., Ltd. (Shanghai, China). Acetic acid was sourced from Macklin Chemical Technology Co., Ltd. (Shanghai, China). Triton X-100 was obtained from Beijing Solarbio Technology Co., Ltd. (Beijing, China). Fresh bovine tendon was obtained from Wuchang Slaughterhouse (Wuhan, China). All of the other commercial reagents and solvents were used without further purification.

### 4.2. Decellularized Extracellular Matrix (dECM) Preparation

The preparation of decellularized extracellular matrix (dECM) was carried out as described in our previous works [38,39]. Briefly, the fresh bovine Achilles tendon tissue was collected, rinsed with sterile saline to remove surface blood and impurities; the surface fat, fascia, and loose connective tissue were carefully removed before cutting the tendon into small pieces of approximately 1 cm × 1 cm. The tissue fragments were placed in a sterile beaker and immersed in a mixed decellularization solution containing 2 wt.% Triton X-100 (TX-100) and 1 wt.% sodium dodecyl sulfate (SDS) at a solid-to-liquid ratio of 1:20 (g:mL), followed by magnetic stirring (1000 rpm) at room temperature (RT) for 24 h (solution was refreshed every 6 h). After decellularization, the samples were thoroughly rinsed with sterile deionized water (30 min per rinse, 6 times in total) to remove residual detergents. The tissues were then subjected to the cycled freeze–thaw process, consisting of freezing at −80 °C for 12 h and thawing in a 37 °C constant-temperature water bath for 12 h, and the freeze–thaw process was repeated 5 times to further disrupt cellular components. Subsequently, the tissues were immersed in 1% (*v*/*v*) acetic acid solution (1:20 g/mL) and stirred (800 rpm) at 4 °C for 36 h, followed by extensive washing with sterile deionized water (water was replaced every 30 min) until the pH was adjusted to the range of 6.8–7.2. The neutralized tissues were then homogenized with an appropriate amount of sterile deionized water using a high-speed homogenizer (10,000–12,000 r/min, 30 s per cycle, 30 s intervals, 3–5 cycles) to obtain a viscous dECM suspension. The solid content of the suspension was measured by the drying method, and the dECM concentration was adjusted to 0.8 wt% by adding sterile deionized water under high-speed stirring. Finally, the prepared dECM suspension was freeze-dried and stored in a desiccator at 4 °C for subsequent use.

### 4.3. dECM@QCS Composite Hydrogels Fabrication

Quaternized ammonium chitosan (QCS) was synthesized by reacting chitosan with GTMAC according to our previous works [38,40]. The synthesized QCS with the substitution degree of the quaternary ammonium group at 4.85 ± 0.42% was used in this work. The dECM powder and QCS solution (1.5 wt.%) were mixed at different mass ratios (QCS/dECM using 1/6, 1/3, 1/2, and 2/3) and dissolved in an appropriate amount of deionized water to form a homogeneous mixture. After thorough stirring, EDC (45 mM) and NHS (25 mM) were added to initiate the crosslinking reaction. The resulting solution was then poured into customized molds and pre-frozen at −80 °C for 24 h. Subsequently, the samples were transferred to a freeze dryer and lyophilized at −50 °C and 0.01 mbar for 48 h, yielding a series of dECM@QCS composite hydrogels (Table 1).

### 4.4. Isolation and Cultivation of Skin-Derived Precursor Cells (SKPs)

The isolation and cultivation of skin-derived precursor cells (SKPs) was accomplished according to the published works [41,42,43]. Animal experiments were approved from the Animal Ethics Committee of Wuhan Myhalic Biotechnology Co., Ltd. (Approval ID: HLK-20250718-001). Sprague-Dawley (SD) rats aged 2 to 3 days were selected as the cell source and euthanized via carbon dioxide inhalation, in compliance with international ethical standards. Briefly, the dorsal skin tissue was promptly collected, and rinsed using PBS buffer multiple times (5–8 times). The rinsed skin tissue was finely minced into approximately 1 mm^3^ pieces. The tissue fragments were then digested with collagenase. After digestion, the tissue pieces were mechanically dissociated into a single-cell suspension by repeated pipetting using polished Pasteur pipettes. Subsequently, the cell suspension was filtered through a 40 μm cell strainer to remove tissue debris and undigested residues, followed by centrifugation at 1000 rpm for 5 min. Subsequently, the cells were resuspended in DMEM/F-12 medium (Gibco, Brooklyn, NY, USA) and seeded into 6-well plates at a density of 1.0 × 10^6^ cells/mL. The culture medium was supplemented with basic fibroblast growth factor (bFGF, 10 ng/mL, Cybio, Woburn, MA, USA), epidermal growth factor (EGF, 20 ng/mL, Cybio, Woburn, MA, USA), and B27 supplement (1: 50, Gibco, Brooklyn, NY, USA). Finally, the cells were cultured at 37 °C and 5% CO_2_, with the culture medium replaced every 3 days to ensure optimal cell growth.

### 4.5. Isolation and Identification of Exosomes

SKPs were cultured in serum-free medium (SFM) supplemented with bFGF and EGF, and when cell confluency reached over 80%, the medium was replaced with fresh SFM. Then, the cells were further cultured for 48 h to collect conditioned medium. The conditioned medium was sequentially subjected to low-speed centrifugation (300 *g*, 5 min; 2000 *g*, 10 min) to remove dead cells and debris, followed by filtration through a 0.22 µm filter. The filtrate was concentrated via ultrafiltration centrifugation (4000 *g*, 30 min) and further purified by ultracentrifugation at 120,000 *g* for 70 min. The purified exosomes were resuspended in PBS, aliquoted, and stored at −80 °C. Particle size distribution and concentration were measured using the NanoSight system, and protein content was quantified using the micro-BCA assay kit (Thermo Fisher Scientific, Waltham, MA, USA). The marker proteins, including CD9, CD63, and CD81, on the surface of the obtained exosomes were expressed using Western blot. Furthermore, Dil/Phalloidin immunofluorescent staining was employed to detect the uptake of exosomes [43,44].

### 4.6. Fabrication of dECM@QCS Hydrogel Encapsulated with Exosomes (exo@dECM@QCS)

Based on the physicochemical property evaluation of dECM@QCS composite hydrogels, sample dECM@QCS 3 hydrogel was selected to encapsulate the obtained exosomes. The prepared dECM@QCS 3 hydrogel was sterilized by ultraviolet irradiation and then immersed in an exosome suspension at a predetermined concentration (100 μg/mL). The hydrogel was incubated at 4 °C for 12 h to allow sufficient exosome adsorption within the porous structure of hydrogel. After incubation, the hydrogel was gently rinsed with PBS to remove loosely bound exosomes. Finally, the exo@dECM@QCS composite hydrogel was obtained and stored at 4 °C for subsequent experiments.

### 4.7. Scanning Electron Microscopy (SEM) Observation

The morphology and microstructure of the dECM@QCS composite hydrogels were characterized by cold field-emission scanning electron microscopy (SEM, Chiyoda-ku, Tokyo, Japan; Hitachi S-4800) investigation [45]. The dECM@QCS composite hydrogels were cut into approximately 1 cm × 1 cm (length × width) pieces and fixed to the sample stage using conductive adhesive. The samples were then gold-sputtered (sputtering current 15 mA, time 60 s) to improve their electrical conductivity. The SEM images of the hydrogels were captured using a scanning electron microscope at an accelerating voltage of 10 kV.

### 4.8. Fourier Transform Infrared Spectroscopy (FT-IR) Investigation

Fourier transform infrared spectroscopy (FT-IR) was used to characterize the functional groups of chitosan, QCS, and dECM@QCS composite hydrogels [46]. Approximately 0.002 g of tested sample was mixed with 0.2 g of pre-dried potassium bromide (KBr) powder. Then the mixture was thoroughly ground in a mortar until uniform and fine, avoiding moisture absorption, after which it was evenly spread in a pellet die, slowly pressed to 10–15 MPa, and held for 60–90 s to prepare transparent, crack-free, uniform, and compact KBr pellets. Subsequently, FT-IR spectra of chitosan, QCS, and dECM@QCS composite hydrogels were collected using FT-IR (Bruker, Bremen, Germany, VERTEX70) in a dry environment with a relative humidity below 50%, using a blank KBr pellet as the background. The wavenumber range of FT-IR spectra was set at 4000–500 cm^−1^ with a resolution of 4 cm^−1^ and 32 scans per measurement.

### 4.9. Equilibrium Swelling Ratio Measurement

The equilibrium swelling ratio (*ESR*) of dECM@QCS composite hydrogels was measured in phosphate-buffered saline (PBS, pH 7.4, Hyclone, Logan, UT, USA). The tested dECM@QCS hydrogel samples were first dried to a constant weight, and the initial weight (*W*_0_) was recorded. The hydrogels were then immersed in PBS solution and incubated at 37 °C. After reaching swelling equilibrium state (around 8 h), the samples were taken out, and the excess surface solution was gently blotted with filter paper, and the weight was measured as *W_t_*. *ESR* of the dECM@QCS composite hydrogels was calculated using the following Equation (1):

(1) $$ESR\;\left(\%\right)=\frac{W_{t}-W_{0}}{W_{0}}\times 100\%$$

where *W*_0_ and *W_t_* represent the initial mass of the dried composite hydrogel and the mass of the composite hydrogel at swelling equilibrium state, respectively.

### 4.10. Degradation Profiles Investigation

The degradation performance of the dECM@QCS composite hydrogels was investigated in SBF using a gravimetric method [47]. The dECM@QCS composite hydrogels were first dried to a constant weight, and the initial mass (*M*_0_) was recorded. The hydrogels were then immersed in simulated body fluid (SBF, pH = 7.4) and incubated in a shaking incubator at 37 °C with a shaking speed of 100 rpm. At the pre-determined days, the hydrogels were removed, rinsed with deionized water, and freeze-dried to a constant weight to obtain the remaining mass (*M_t_*). The degradation degree (*DD*) of the dECM@QCS composite hydrogels was calculated using the following Equation (2):

(2) $$DD\;\left(\%\right)=\frac{M_{t}-M_{0}}{M_{0}}\times 100\%$$

where *M*_0_ represents the initial weight of the composite hydrogels, and *M_t_* represents the weight of the composite hydrogels after freeze-drying at different time points.

### 4.11. Cytocompatibility Evaluation

The cytocompatibility of dECM@QCS and exo@dECM@QCS composite hydrogels was investigated by culturing with mouse embryonic fibroblast cells (NIH 3T3, ATCC, CRL-1658™, Manassas, VA, USA) using fluorescent images and Cell Counting Kit-8 (CCK-8) assay [48]. According to the ATCC instructions, NIH 3T3 cells were cultured in complete medium (10 *v*/*v*% fetal bovine serum, 1 *v*/*v*% penicillin-streptomycin, 89 *v*/*v*% DMEM) at 37 °C in a humidified atmosphere containing 5% CO_2_. After resuscitation, NIH 3T3 cells were cultured to the logarithmic growth phase and seeded into 96-well plates at a density of 5 × 10^3^ cells/well. The cells were pre-cultured at 37 °C with 5% CO_2_ for 24 h until reaching 70–80% confluency. Subsequently, the cells were gently washed using PBS, and the medium was replaced with serum-free medium containing hydrogel extracts. Then, the cells were cultured for 1, 3, and 5 days, 10 µL of CCK-8 reagent was added to each well, the plates were gently shaken to mix, and the plates were incubated in the dark at 37 °C with 5% CO_2_ for 4 h. Finally, the optical density (*OD*_450_) value was measured using a microplate reader to measure the cell viability. In addition, the cell morphology was observed using Calcein-AM (live cells) and propidium iodide (dead cells) staining treatment at day 1 and 3, and the fluorescent images were captured using an inverted fluorescence microscope.

### 4.12. Cell Scratch Assay

The effect of the SKP-derived exosomes in exo@dECM@QCS composite hydrogels on promoting cell migration was investigated using a scratch wound assay [48]. NIH 3T3 cells were seeded in 6-well plates at a density of 5 × 10^5^ cells/well and cultured at 37 °C for 24 h to allow for cell adhesion and growth. After the cells reached full confluence, a sterile 200 μL pipette tip was used to create a uniform linear scratch by vertically dragging it along the well’s central axis. The cells were then gently washed 3 times with PBS to remove detached debris. After discarding PBS, the samples were incubated in serum-free medium containing biomaterial extracts and SKP-derived exosome suspension for 24 h. Subsequently, the scratched regions were observed and photographed using an inverted microscope, and the images were quantitatively analyzed with ImageJ software (Version 1.53). Furthermore, the human umbilical vein endothelial cells (HUVECs, Chinese Academy of Sciences, Shanghai, China) were also used to investigate the cell pro-migration ability of exosomes and composite hydrogels using the above procedure.

### 4.13. Antibacterial Activity Investigation

The typical Gram-positive *Staphylococcus aureus* (*S. aureus*, ATCC 6538) and Gram-negative *Escherichia coli* (*E. coli*, ATCC 25922) were used to evaluate the antibacterial properties of the dECM@QCS and exo@dECM@QCS composite hydrogels according to the previous reported work [49]. First, a logarithmic growth phase bacterial suspension with a concentration of 1 × 10^6^ CFU/mL was prepared. This suspension was then co-cultured with the dECM@QCS and exo@dECM@QCS composite hydrogels at 37 °C for 24 h. The samples were then directly subjected to spread plate culture for 24 h to observe colony formation.

### 4.14. Macrophages Phenotypic Transition Evaluation

The influence of the dECM@QCS and exo@dECM@QCS composite hydrogels on the transition of macrophage phenotypes was evaluated using immunofluorescence staining. Briefly, RAW 264.7 cells were seeded into a 6-well plate (1 × 10^6^ per well) for 24 h. Next, the cells were treated using LPS (50 ng/mL) for additional 12 h. Then, the hydrogel extracts were added and cultured for 12 h. The group with adding PBS as the control group. The cells were stained using primary antibodies (Abcam, Cambridge, UK), including iNOS was diluted at 1:50, and Arg1 was diluted at 1:100. The expression of M1-type marker iNOS (green) and M2-type marker Arg1 (red) in macrophages were investigated by capturing the fluorescent images using an inverted fluorescence microscope. Furthermore, the fluorescence intensities of iNOS and Arg1 were measured.

### 4.15. Intracellular Reactive Oxygen Species (ROS) Levels Evaluation

The intracellular levels of reactive oxygen species (ROS) in HUVECs influenced by the dECM@QCS and exo@dECM@QCS composite hydrogels were determined using the DCFH-DA fluorescent probe [24]. Hydrogen peroxide (H_2_O_2_) was diluted in DMEM to prepare H_2_O_2_ solution with a final concentration of 100 μM. HUVECs were treated with 1 mL of H_2_O_2_ solution supplemented with 10 μL of hydrogel extracts. Without adding hydrogel extracts as the positive control group, and the 1 mL of complete culture medium alone as the blank control group. All groups were then incubated in the dark at 37 °C for 2 h to establish oxidative stress. Then, the solutions were removed, and the cells were washed 3 times with PBS to eliminate residual H_2_O_2_ and hydrogel extracts completely. The DCFH-DA probe was diluted 1:1000 in serum-free medium, and 1 mL of the diluted DCFH-DA solution was added to each dish to fully cover the cell monolayer, followed by incubation in the dark at 37 °C for additional 30 min. The cells were then washed 3 times with PBS, and the fluorescent images were acquired using an inverted fluorescence microscope.

### 4.16. Transwell Assay

The pro-angiogenic capacity of SKP-derived exosomes and composite hydrogels was investigated using the Transwell assay according to the previously reported method [48]. HUVECs were seeded into the upper chambers of Transwell inserts (5 × 10^4^ cells/well in serum-free medium) placed in a 24-well plate. The lower chambers were filled with complete medium containing 10% fetal bovine serum (FBS) to act as a chemoattractant. Experimental groups were treated with the serum-free medium containing hydrogels extracts and SKP-derived exosomes, while untreated HUVECs served as the control group. After incubation at 37 °C for 48 h, cells were washed with PBS, fixed with 4% paraformaldehyde for 30 min, and stained with 0.1% crystal violet for 10 min. Images were captured using an inverted microscope, and the number of transmigrated cells was quantified.

### 4.17. EdU Assay

EdU incorporation assay was used to assess DNA synthesis in HUVECs, thereby evaluating the regulatory effect of skin-derived precursor cell exosomes and composite hydrogels on the proliferative capacity of HUVECs [50]. HUVECs were seeded into 24-well plates at a density of 5 × 10^4^ cells per well and cultured until reaching 60–70% confluency. EdU solution was then added to each well, followed by incubation at 37 °C for 4 h to allow incorporation of EdU into newly synthesized DNA. After incubation, HUVECs were fixed with 4% paraformaldehyde for 20 min at room temperature, followed by permeabilization with 0.3% Triton X-100 for 15 min. HUVECs were then incubated in the dark for 30 min. Nuclei were counterstained with DAPI for 5 min. Finally, the fluorescent images were captured using a fluorescence microscope (Nikon, Tokyo, Japan), and the proportion of EdU-positive cells was quantified using ImageJ software to evaluate cell proliferation ability.

### 4.18. Tube Formation Assay

An in vitro tube formation assay was performed to evaluate the pro-angiogenic effect of SKP-Exos and composite hydrogels on HUVECs according to the previous work [51]. Pre-chilled 96-well plates were coated with 60 μL of Matrigel per well, then gently swirled to ensure uniform coverage. The plates were then incubated at 37 °C with 5% CO_2_ for 1 h to allow Matrigel to solidify. HUVECs in logarithmic growth phase were resuspended in serum-free medium at a density of 2 × 10^5^ cells/mL, followed by supplementation with medium containing hydrogels extracts and SKP-derived exosomes. After thorough mixing, 100 μL of the cell suspension was added to each Matrigel-coated well and incubated for 6 h. Following incubation, tubular structures formed by HUVECs were photographed under an inverted microscope.

### 4.19. In Vivo Animal Experiments

This experiment aims to investigate the wound healing effects of SKP-Exos and composite hydrogels in diabetic rats, providing experimental evidence and theoretical support for the development of effective therapeutic strategies for diabetic skin defects [52]. In vivo animal experiments were approved from the Animal Ethics Committee of Wuhan Myhalic Biotechnology Co., Ltd. (Approval ID: HLK-20250718-001). The animal experiments conformed to the “3R” principle of laboratory animals, and strictly complied with the guidelines for the use and management of laboratory animals issued by the National Health and Family Planning Commission of China. Streptozotocin (100 mg/kg, dissolved in 0.01 mol/L sodium citrate buffer, pH = 4.3) was injected intraperitoneally into rats to establish a diabetic rat model. Based on changes in blood glucose levels, insulin was injected into the rats to maintain blood glucose levels within the range of 16.7–33.3 mM per liter. The model was confirmed to be successful after three consecutive days. Four weeks later, the full-thickness skin defects with a diameter of 15 mm were created using sterile surgical shears. The rats were randomly divided into four groups: the blank control group, dECM@QCS group, the exos group, and the exo@dECM@QCS group. Wound tissue samples were collected on days 3, 7 and 14, post-treatment, for subsequent wound healing evaluation and analysis.

### 4.20. Histological and Immunohistochemical Staining Analysis

Through multiple tissue staining methods combined with image analysis, the effects of skin-derived precursor cell exosomes and the composite hydrogels on wound healing were further verified, providing tissue-level evidence for tissue repair and regeneration [53]. The collected tissue samples were fixed in 4% paraformaldehyde for 24 h, followed by paraffin embedding according to standard histological protocols. The embedded tissues were sectioned into 4–6 μm thick slices, which were then subjected to hematoxylin and eosin (H&E) staining to observe tissue morphology, Masson’s trichrome (MT) staining to evaluate the distribution and density of collagen fibers. Immunofluorescence staining was performed to detect the expression and localization of specific proteins. On day 3 post-treatment, the expression of M1/M2 macrophage polarization markers CD86 and CD206 was evaluated. On day 14, the expression of α-SMA and CD31 was assessed to determine fibroblast activation and angiogenesis levels. Finally, the samples were observed under an optical microscope, and images were captured. Image analysis software was utilized to perform histological examination, quantitative statistics, and qualitative analysis, thereby comprehensively assessing tissue repair and regeneration.

### 4.21. Statistical Analysis

All trials were conducted in triplicate, with results expressed as mean ± SD (standard error of the mean). Statistical comparisons were performed through one-way ANOVA using IBM SPSS Statistics (Version 22), followed by post hoc LSD testing for intergroup comparisons. “ns” indicates no statistical difference, * *p* < 0.05, ** *p* < 0.01, and *** *p* < 0.001 vs. control.

## Figures and Tables

**Table 1:** The composition of QCS, dECM for the fabrication of dECM@QCS composite hydrogels.

Sample	QCS	dECM	EDC/NHS
dECM/QCS 1	0.3 g	0.05 g	0.14/0.06 g
dECM/QCS 2	0.3 g	0.1 g	0.14/0.06 g
dECM/QCS 3	0.3 g	0.15 g	0.14/0.06 g
dECM/QCS 4	0.3 g	0.2 g	0.14/0.06 g

## Data Availability

The data presented in this study are available on request from the corresponding author. The data are not publicly available due to ethical.
